# Longitudinal Analysis of Variations in Daily Step Counts and Long-Term Implications of COVID-19 Waves and Restriction Phases in Qatar’s Step Into Health Program: Mixed Methods Study

**DOI:** 10.2196/76860

**Published:** 2026-03-23

**Authors:** Lina Majed, Suzan Sayegh, Feriel Dalansi, Abdulla Saeed Al-Mohannadi, Marco Cardinale, Abdulaziz Farooq

**Affiliations:** 1 College of Health and Life Sciences Hamad bin Khalifa University Doha Qatar; 2 Faculty of Health Sciences Modern University for Business and Science Beirut Lebanon; 3 Shift Wellness Institute Vancouver, WA United States; 4 Research and Scientific Support Aspetar, Orthopaedic and Sports Medicine Hospital Doha Qatar; 5 Department of Orthopedic Surgery and Sports Medicine Amsterdam Movement Sciences, Amsterdam UMC University of Amsterdam Amsterdam The Netherlands; 6 World Innovation Summit for Health (WISH) Qatar Foundation Doha Qatar; 7 Institute of Sport Exercise and Health University College London London United Kingdom; 8 Department of Sport Exercise and Rehabilitation Northumbria University Newcastle Upon Tyne United Kingdom

**Keywords:** community-based intervention, coronavirus disease, COVID-19, interviews, lockdown, longitudinal data, mixed methods, mobile app, pandemic, pedometer, step count

## Abstract

**Background:**

Public health restrictive measures adopted during the COVID-19 pandemic led to significant changes in lifestyles. Global declines in physical activity (PA) and increases in sedentary behavior were noted. These trends were observed within different regions of the world, pointing toward potential long-term implications for PA behaviors.

**Objective:**

This mixed methods study aims to assess variations in daily step counts in Qatar using device-driven data throughout all 3 COVID-19 waves (February 2020 to February 2023) compared with a full pre–COVID-19 year. In-depth interviews were further conducted with randomly selected participants to gain insights into determinants, perceptions, and barriers of PA during the pandemic.

**Methods:**

A total of 362 participants (60/362, 16.6% female) from the Step Into Health community-based program reported daily step counts using pedometers (170/362, 47%) or a mobile phone app (192/362, 53%). Linear mixed models examined changes in daily step counts across 19 phases of implementation and lifting of restrictions. Overall, 9 participants also completed semistructured interviews that were analyzed thematically and phenomenologically. Triangulation of quantitative and qualitative data was applied to interpret convergences and divergences between device-measured activity patterns and lived experiences.

**Results:**

Significant declines in daily step counts (ie, from 689 to 1013 steps) were observed at the onset of each wave (*P*<.001), were especially marked at wave 2, and were followed by a recovery of step count following the lifting of restrictions at each wave (ie, increase of 609 to 1147 steps). Different patterns of change in step count emerged within sex (*P*=.03), age (*P*=.03), and BMI (*P*=.01) groups, where larger variations were seen among male individuals, pedometer users, and normal-weight participants. Qualitative themes (ie, disrupted routines, reliance on home-based exercise, and media influence) contextualized these patterns and explained subgroup differences.

**Conclusions:**

The largest drops in daily step count coincided with increased case severity and Ramadan. Integration of quantitative and qualitative findings showed that declines in activity were shaped not only by restrictions but also by fear, motivation, and contextual factors. These results underscore the importance of designing interventions that encourage outdoor activity and provide reliable social media–based guidance during public health crises.

## Introduction

COVID-19 was initially detected in December 2019 in Wuhan, China. This disease is caused by SARS-CoV-2 [1]. On March 11, 2020, the World Health Organization declared COVID-19 a global pandemic [2]. The first confirmed case of COVID-19 in Qatar was reported on February 27, 2020 [3]. Since then, progressive restrictions have been implemented in Qatar and worldwide as preventive measures to reduce the spread of the virus. These measures targeted various sectors, including business and leisure, transportation, outdoor and professional sports, education, health services, and social gatherings.

Physical activity (PA) is a critical determinant of health, contributing to the prevention of chronic diseases and the promotion of overall well-being [4]. According to the World Health Organization, adults aged 18-64 years are recommended to engage in at least 150-300 minutes per week of moderate-intensity PA, or a minimum of 75-150 minutes per week of vigorous-intensity PA, or a combination of both for substantial health benefits [5]. However, fulfilling these recommendations became particularly challenging during COVID-19 restrictions, although many countries have published targeted PA guidelines for times of pandemics, outbreaks, and home quarantine (eg, Qatar National PA Guidelines [6]). A global decline in total PA levels was observed [7-11] including vigorous-, moderate-, and light-intensity PA [8,9,12-14]. This was accompanied by a significant increase in total sedentary time [15,16].

Adequate and consistent PA has been suggested to enhance immune responses against respiratory viral infections, emphasizing its relevance during the pandemic [17]. Reductions in PA, along with increased sedentary behavior, can lead to adverse health outcomes such as diminished physical fitness and an impaired immune system response, negatively affecting quality of life [4].

In Qatar, a study examining PA levels before and during the first lockdown phase in 2020 revealed significant reductions in walking and both vigorous and moderate PA levels and increases in sedentary behavior [18]. A general decline in life satisfaction was also noted and reflected the profound physical and mental impact of the imposed restrictions [18]. Another survey-based study on Qatar’s adult population confirmed increases in sedentary time and decreases in exercise time, which were associated with gains in body weight [19]. Both studies presented a few limitations, such as the reliance on subjective self-reported data and the lack of comparison with other periods of reduced PA in Qatar (eg, summer months or Ramadan). To address the latter, a recent study explored daily variations in step count before, during, and after the first COVID-19 wave in Qatar, covering a period of approximately 1 year [20]. Authors showed that the significant declines in daily step count following restrictions persisted after the lifting of restrictive measures, affecting mostly those who were physically active at baseline (ie, men, normal weight, and older individuals) [20]. The COVID-19 restrictions, which aimed to curb the spread of the virus, have continued disrupting normal life patterns in Qatar until November 2022. However, none of the studies from Qatar accounted for the second and third COVID-19 waves that resulted in potential long-term implications to PA. Such longitudinal data could provide better insights into the dynamics of PA behaviors in times of extended public health safety measures, particularly in regions where there is a paucity of data on PA changes during pandemics.

Lockdowns in the Arabian Gulf Peninsula and the Middle East and North Africa regions inadvertently decreased opportunities for regular PA and exacerbated sedentary lifestyles [21,22]. Although restrictions have been lifted, the long-term effects of the COVID-19 pandemic on physical and mental health are still emerging [23,24]. While much has been done on the immediate impacts, long-term behavioral shifts present a critical gap, particularly in regions like the Gulf Peninsula region, with its unique environmental and cultural characteristics [25].

Given the challenges and potential biases present in assessing PA through self-reported data and the rather short-term nature of most studies in the region, this study seeks to explore the impact of all 3 waves of COVID-19–related restrictions on device-measured daily step counts in Qatar. Understanding these disruptions over a period of 3 years is vital for shaping public health strategies that promote active lifestyles and address the ongoing post–COVID-19 challenges [26]. Using a mixed method approach, this study further aims to assess associated barriers, behavior changes, attitudes, and perceptions during the pandemic, providing valuable insights into how restrictions have influenced PA patterns in Qatar and potentially the region.

## Methods

### Ethical Considerations

All participants in the “Step Into Health” program (SIH) provided a disclaimer at enrollment permitting the use of their step count data for research and program evaluation. Only limited, deidentified data were extracted from the SIH database for this study. For the qualitative part, interview participants provided separate written informed consent, including permission to audio-record the interview and to use anonymized quotations in publications. No compensation or incentives were provided to participants for their involvement in this research. Privacy and confidentiality were strictly maintained in accordance with institutional review board approval (E202104021).

### Study Design and Population

This is a retrospective longitudinal cohort study, which aimed to explore the impact of all waves of COVID-19–related restrictions in Qatar on PA levels using mixed methods. Participants included in this research were members of the SIH, a pedometer-based community intervention designed to encourage residents of Qatar to walk 10,000 steps per day [20,27-30]. SIH is the largest PA community-based intervention in Qatar and is still ongoing since 2012. The program was initially publicized nationally through outreach advertisement campaigns targeting different settings (workplaces, campuses, and malls). Registration to the SIH was done on a voluntary basis through a dedicated online platform [20,27-30]. Upon registration, participants received a free pocket-sized pedometer (Omron HJ-324U, Omron Healthcare Co, Ltd) or a mobile phone app (Android and iOS operating systems; SIH; version 3.0.3; Aspire Zone), both of which were linked to a web database that recorded step counts [20,27-30].

Male and female participants of all nationalities (as registered in the SIH database) were included in the analysis. From the initial pool of participants that were actively recording their step count during the studied time frame (January 2019 to November 2022), a total of 3562 participants were retained after excluding those aged 18 years or younger or those presenting invalid step counts (ie, missing data or less than 500 or more than 60,000 steps per day). A further filtering step excluded participants presenting less than 3 activity days per week [31] or 5 days per month [32] over the entire studied period. After applying the last step, 362 participants were retained for the quantitative analysis.

### Qualitative Research

From the retained sample, 9 participants were invited to participate in in-depth interviews. A random selection process was applied (ie, every 40th individual in the SIH database) to reduce researcher selection bias and give all participants an equal chance of recruitment. Although not purposive, this process resulted in a sample with diversity in sex, age, and activity behaviors, which allowed a range of perspectives to be captured. Data collection continued until thematic saturation was observed, with no new themes and codes emerging after the ninth interview. At that point, the information became repetitive across participants, indicating that the sample size was sufficient to capture the diversity of perspectives. Participants were contacted individually by phone to confirm their participation. Interviews occurred between September 2021 and November 2021.

### Study Measures

#### Quantitative: Daily Step Count

The mobile phone app reads steps counted by the phone’s built-in sensors and transfers the data to the cloud. Pedometer users were asked to upload their data daily or as frequently as possible. More information on the program is cited elsewhere [20,27-30].

Data extracted from the SIH web database included the available active members who had uploaded their steps before, during, and after the COVID-19–related restrictions phases. Demographic data, self-reported upon registration, were extracted from participants’ SIH profiles, which included age, sex, and nationality, as well as body height and mass. BMI was calculated as the ratio between mass (kg) and height squared (m^2^). Participants were then grouped according to their sex (ie, male and female), age (ie, <50 years and ≥50 years), nationality (ie, Qatari and non-Qatari), BMI classification (ie, normal weight with values <25, overweight with values between 25 and <30, and obesity with values of ≥30) and the type of device used (ie, pedometer or mobile phone app).

#### Qualitative: In-Depth Interviews

This study used qualitative methods to explore participants’ experiences, attitudes, and perceptions regarding PA during the COVID-19 pandemic in Qatar. A total of 9 in-depth interviews were conducted remotely via online platforms or phone calls with participants of the SIH program to examine behavioral changes, barriers, and motivators related to PA during all restrictive waves of the COVID-19 pandemic. The sample included 5 male and 4 female individuals, selected to ensure diverse perspectives.

All interviews were conducted in English or Arabic, based on the participant’s preference. Interviewers were fluent in both languages, and interview questions were translated into Arabic for Arabic-speaking participants. Prior to the interviews, interviewers received comprehensive training on the interview protocol to ensure consistency and clarity in question delivery.

The interviews were semistructured, focusing on key themes such as the importance of PA, changes in exercise habits prepandemic and postpandemic, the impact of COVID-19 restrictions on PA, barriers to staying active, and motivators for exercise. Participants were also asked about their experiences with home-based exercise routines, the role of the media in shaping their activity choices, and their intentions regarding future PA post restrictions.

Each interview lasted between 30 and 60 minutes. Interviews were conducted virtually or in-person, adhering to the appropriate COVID-19 safety protocols for in-person sessions. All interviews were audio-recorded for quality assurance, and transcripts were created from the recordings. Deidentified transcripts were stored for record-keeping and coding purposes.

### Timeline of the 3 COVID-19 Waves in Qatar

Table 1 details the main COVID-19 phases considered during the 3 waves relating mainly to the restrictions that were imposed and lifted by the Qatar’s Ministry of Public Health. A pre–COVID-19 period of over a full year (T0) is used as a reference to annual changes in daily step count. The data presented in Table 1 were compiled from the Ministry of Public Health website and other reliable news sources from the written press.

**Table 1:** Timeline of implementation and lifting of COVID-19 restrictions during the 3 COVID-19 waves in Qatar. T0 represents a pre–COVID-19 reference phase of more than 1 year.

Phase	Start date	End date	Description
T0	January 1, 2019	February 28, 2020	Pre–COVID-19
T1	February 29, 2020	March 11, 2020	First confirmed case in Qatar until the start of lockdown
T2	March 12, 2020	June 14, 2020	Wave 1: Qatar begins to implement lockdowns
T3	June 15, 2020	June 30, 2020	Wave 1: Phase 1 gradual lifting of restrictions
T4	July 1, 2020	July 31, 2020	Wave 1: Phase 2 gradual lifting of restrictions
T5	August 1, 2020	August 31, 2020	Wave 1: Phase 3 gradual lifting of restrictions
T6	September 1, 2020	February 3, 2021	Wave 1: Phase 4 gradual lifting of restrictions
T7	February 4, 2021	May 27, 2021	Wave 2: Qatar to reimpose COVID-19 restrictions
T8	May 28, 2021	June 17, 2021	Wave 2: Phase 1 gradual lifting of restrictions
T9	June 18, 2021	July 8, 2021	Wave 2: Phase 2 gradual lifting of restrictions
T10	July 9, 2021	August 5, 2021	Wave 2: Phase 3 gradual lifting of restrictions
T11	August 6, 2021	October 2, 2021	Wave 2: Phase 3.2 gradual lifting of restrictions
T12	October 3, 2021	February 1, 2022	Wave 2: Phase 4 gradual lifting of restrictions
T13	February 2, 2022	April 1, 2022	Wave 3: Qatar reimposes COVID-19 restrictions
T14	April 2, 2022	May 20, 2022	Wave 3: gradual lifting of restrictions: 100% employees back to work
T15	May 21, 2022	July 6, 2022	Wave 3: gradual lifting of restrictions: mask required in health care
T16	July 7, 2022	August 31, 2022	Wave 3: reimpose mask in all settings
T17	September 1, 2022	October 31, 2022	Wave 3: gradual lifting of restrictions: mask required in health care
T18	November 1, 2022	February 28, 2023	Wave 3: gradual lifting of restrictions: Ehteraz^a^ no longer required

^a^Ehteraz is the mandatory mobile app used in Qatar to track the population’s health status during COVID-19.

### Statistical Analysis

#### Quantitative Analysis

Data were analyzed using the SPSS software (version 21.0; IBM Corp). A descriptive analysis presented continuous variables (eg, age and BMI) and daily step count as mean, SD, and 95% upper and lower CIs. Distribution (count and frequencies) was used to present participants’ characteristics on categorical variables like nationality and sex. Time series 7-day moving average on daily step count was computed to plot the trend of PA across the study period stratified by age, sex, BMI status, and nationality. Linear mixed models were used to examine changes in daily step counts over all the 19 studied time phases (Table 1), based on independent factors such as sex, age, BMI classification, nationality, and device. All factors and their interaction with factor time were included in the linear mixed models. Pairwise comparisons were used to examine significant changes in step count between the time phases studied. By adding subject ID as a random effect with a random intercept, we accounted for individual-level variation and within-subject correlation. An unstructured covariance structure was found to be the best fit.

#### Qualitative Analysis

The qualitative data from the in-depth interviews were analyzed thematically to identify key patterns and insights related to participants’ experiences, attitudes, and perceptions of PA during the COVID-19 pandemic. Transcripts of the interviews were reviewed and coded inductively, allowing for the emergence of recurring themes associated with changes in PA behaviors, perceived barriers, and motivators, and the broader psychological and emotional impacts of the pandemic.

The qualitative analysis was conducted by 1 researcher (SS), who has training and experience in qualitative research. A thematic analysis was carried out following Braun and Clarke’s [33] 6-step framework (ie, familiarization with data, coding, generating themes, reviewing themes, defining/naming themes, and reporting). This inductive process enabled the identification of recurring patterns. A phenomenological approach was then used to interpret the lived experiences of participants, focusing on the subjective meaning they attributed to disruptions and adaptations in their PA routines during the pandemic. Although a single researcher conducted the coding, credibility was strengthened through repeated immersion in the transcripts, detailed documentation of coding decisions, and reflective discussions with the wider research team to ensure interpretations were consistent and trustworthy.

To integrate the qualitative and quantitative findings, methodological triangulation was used. Quantitative step count data and qualitative interview data were first analyzed independently, then compared to identify areas of convergence and divergence. Data triangulation was also incorporated by drawing on step count measurements from 2 different devices (pedometers and mobile apps) alongside qualitative accounts. This approach enhanced the depth and validity of the findings by linking device-measured activity with participants’ lived experiences and contextual factors.

## Results

### Quantitative Results

#### Population’s Characteristics

A total of 362 participants, including 60 (16.6%) female and 302 (83.4%) male individuals, met the inclusion criteria, demonstrating consistent tracking of their daily steps throughout the entire pandemic period. On average, participants were aged 47.4 (SD 9.0) years and presented a BMI of 27.9 (SD 4.4) kg/m^2^. About a third of the studied population (n=137, 37.8%) was aged 50 years or older, while 75.1% (272/362) were classified either with overweight (n=184, 50.8%) or obesity (n=88, 24.3%). Qatari nationals represented 30.7% (n=111) of the studied population, which reflects a relatively good representation compared to the overall population of Qatar. From the entire sample, 170 (47%) participants were pedometer users and 192 (53%) used the mobile app (ie, n=58, 16% Android and n=134, 37% Apple iPhone) to track their activity level.

#### Effect of COVID-19 Phases on Step Count

**Figure 1:**
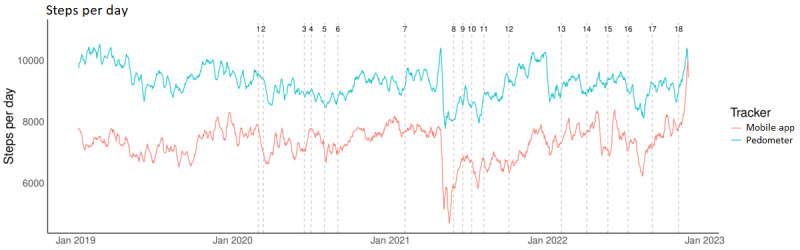
The 7-day moving average of daily step count for pedometer and mobile app users within the studied period covering 1 year before COVID-19 (T0: January 1, 2019, to February 28, 2020) and the 3 COVID-19 waves (T1-T18: February 29, 2020, to February 28, 2023). Details on the 19 time points reflecting the implementation and lifting of restrictions are found in Table 1.

The analyses revealed a significant effect of time (*F*_18, 5027.03_=5.901; *P*<.001), device (*F*_1, 1598.03_=6.275; *P*=.01), and an interaction between device and time (*F*_18, 5157.67_=1.70; *P*=.03) on step count (Figure 1). In comparison to the pre–COVID-19 period, significant decreases in step count ranging from 689 to 1013 steps were observed following implementation of restrictive measures for each of the 3 COVID-19 waves (Figure 1). Specifically, restrictions reimposed at the start of wave 2 brought about the largest decreases in daily step count. With the lifting of restrictions, significant increases in step count were found at the end of each wave (ie, between 609 and 1147 steps; Figure 1). On average, pedometer users presented 690 additional daily steps as compared to those using the mobile app (*P*=.01; Figure 1). Main patterns of change in step count with time were comparable between pedometer and mobile users, with some interactions noted. For instance, after September 2022 (ie, T17 and T18, end of wave 3) the increase in step count among mobile users was larger than that observed in pedometer users with the final lifting of restrictions (Figure 1). In the following sections, data from pedometer and mobile users are presented separately.

#### Effect of COVID-19 Phases on Step Count by Sex and Nationality Groups

**Figure 2:**
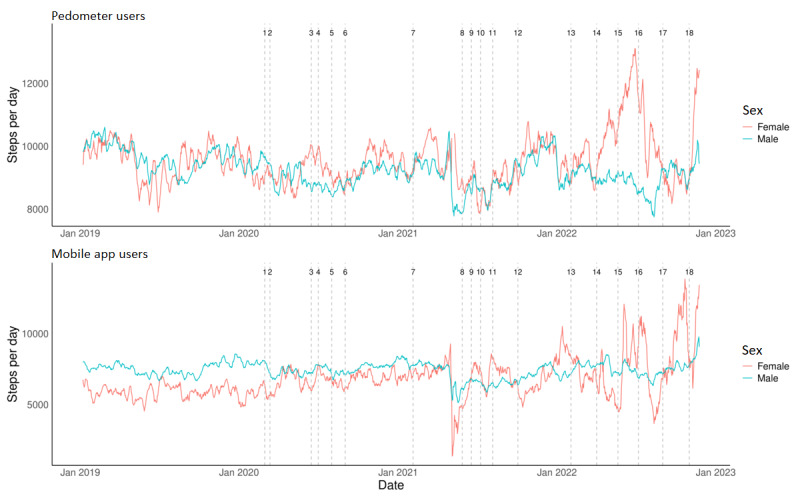
The 7-day moving average of daily step count for male and female groups within the studied period covering 1 year before COVID-19 (T0: January 1, 2019, to February 28, 2020) and the 3 COVID-19 waves (T1-T18: February 29, 2020, to February 28, 2023). Details on the 19 time points reflecting the implementation and lifting of restrictions are found in Table 1. Data from pedometer and mobile app users are presented separately.

Although the main effect of sex (*F*_1, 360.74_=0.125; *P*=.72) and its interaction with device (*F*_1, 2384.61_=0.888; *P*=.35) or time (*F*_18, 5167.25_=1.39; *P*=.12) were not statistically significant, post hoc comparisons revealed noticeable significant sex differences, mostly evident for mobile users (Figure 2). For mobile users (Figure 2), female individuals recorded an average of 1764 fewer daily steps as compared to male individuals at the pre–COVID-19 period (*P*=.03). The only other large difference between male and female groups was detected at the lifting of restrictions in wave 3, which corresponded to the return to work of 100% of the employees in Qatar (T14, 2227 less steps in female compared to male individuals; *P*=.01; Figure 2). However, step count for female mobile users was not significantly affected by the first or second waves of COVID-19, where even significant increases in values were recorded at the end of wave 1 (+1059 steps; *P*=.046) and at the last studied phase (+2219 steps; *P*=.01) compared to pre–COVID-19 (Figure 2). Male mobile users recorded significant decreases in steps at each of the first 2 waves as compared to the pre–COVID-19 period (–932 steps; *P*<.001; –954 steps; *P*<.001; respectively); however, they were less affected by the third wave, where a significant increase in values was observed at the last studied phase compared to pre–COVID-19 (+871 steps; *P*=.008; Figure 2). For pedometer users, sex differences were less evident at the pre–COVID-19 period; however, female individuals recorded 1736 more steps at the time when 100% of employees returned to work during wave 3 (T14; *P*=.05; Figure 2). The only significant change in step count of female pedometer users was seen in wave 2 with a decrease of –1443 steps compared to pre–COVID-19 (*P*=.03), after which step count went back to pre–COVID-19 values (Figure 2). Step count of male pedometer users was more significantly affected by the restrictions imposed during all COVID-19 waves, where significant declines compared to pre–COVID-19 of 993 steps (wave 1; *P*<.001), 1099 steps (wave 2; *P*<.001) and 1499 steps (wave 3; *P*<.001) were observed (Figure 2). Values remained significantly lower throughout wave 3 and were still 708 steps lower than pre–COVID-19 at the last studied phase (*P*=.06).

No significant differences in daily step count were observed between the nationality groups (*P*>.05). Please refer to Tables S2 and S3 in Multimedia Appendix 1 for the average daily step count of each of the 19 studied periods for pedometer and mobile users, respectively, among all subgroups.

#### Effect of COVID-19 Phases on Step Count by Age Group

**Figure 3:**
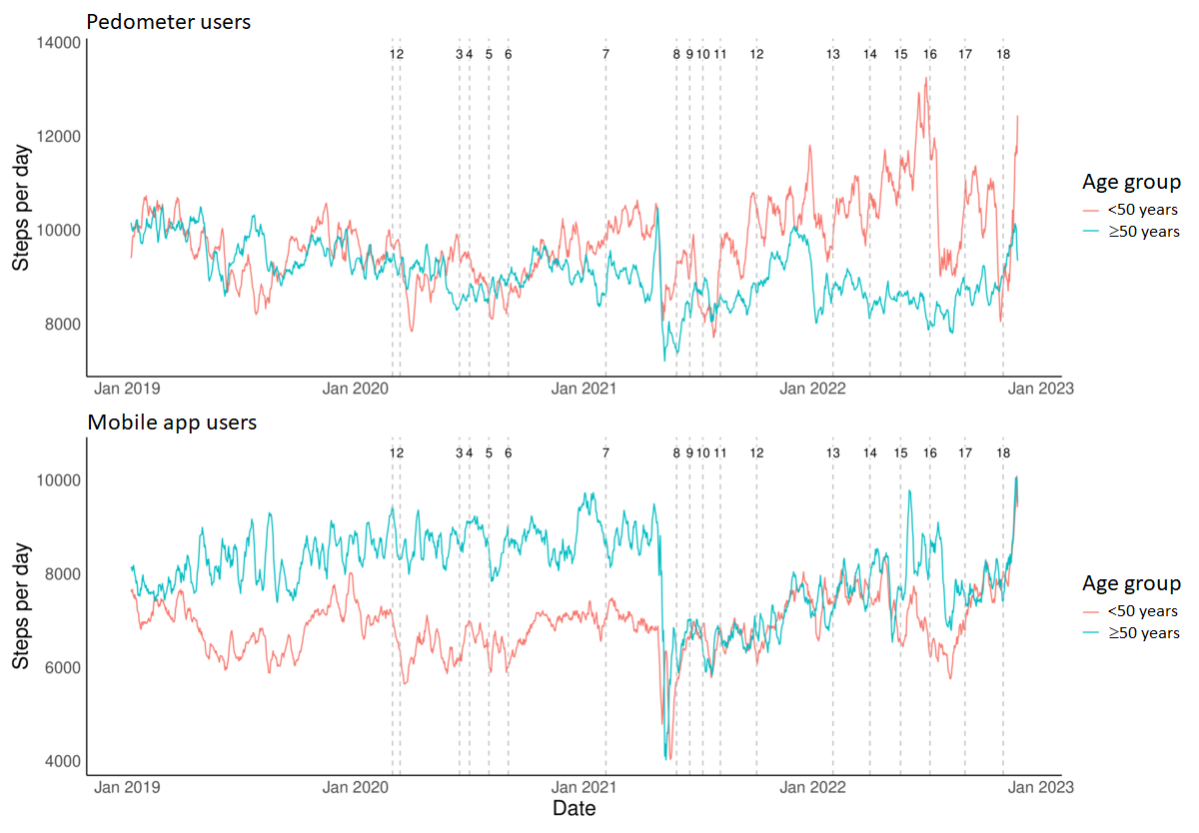
The 7-day moving average of daily step count for the younger (ie, ≤ 50 years) and older (ie, >50 years) groups within the studied period covering 1 year before COVID-19 (T0: January 1, 2019, to February 28, 2020) and the 3 COVID-19 waves (T1-T18: February 29, 2020, to February 28, 2023). Details on the 19 time points reflecting the implementation and lifting of restrictions are found in Table 1. Data from pedometer and mobile app users are presented separately.

A significant effect of age (*F*_1, 9233.62_=4.919; *P*=.03) was evident on daily step count (Figure 3) in the studied period. Generally, the older group (ie, >50 years) moved less, with an average of 301 fewer steps than the younger group (ie, ≤ 50 years; Figure 3). A significant interaction effect between age and device type (*F*_1, 9031.59_=24.764; *P*<.001) and a more detailed post hoc comparison showed a different pattern of change in step count between age groups according to the device used (Figure 3). In mobile users, the older group had initially higher step counts at the pre–COVID-19 period compared to the younger group (1344 steps) and were less affected by the first wave of COVID-19 (Figure 3). Step count of the older group, however, was significantly decreased at the second wave (–1545 steps at T11 as compared to pre–COVID-19; *P*<.001), which persisted through the entire second wave and most of the third wave. At the end of the studied period (T18), both groups regained pre–COVID-19 values, with the younger even presenting 1562 additional daily steps as compared to pre–COVID-19 (Figure 3). For pedometer users, a significant decrease in step count was recorded at the start of wave 1 for younger (–838 steps; *P*=.003) and older groups (–756 steps; *P*=.005). While the younger group’s step count regained pre–COVID-19 values at the end of waves 2 and 3 with gradual increases recorded until the last phase (+699 steps compared to T0), the older group did not regain initial pre–COVID-19 values following restrictions of waves 2 and 3, with further decreases recorded at wave 3 (–1585 steps as compared to pre–COVID-19; *P*<.001) and significantly lower values at the last period (–768 steps) as compared to the pre–COVID-19 period (*P*=.48; Figure 3). Please refer to Tables S2 and S3 in Multimedia Appendix 1 for the average daily step count of each of the 19 studied periods for pedometer and mobile users, respectively, in all subgroups.

#### Effect of COVID-19 Phases on Step Count by BMI Groups

**Figure 4:**
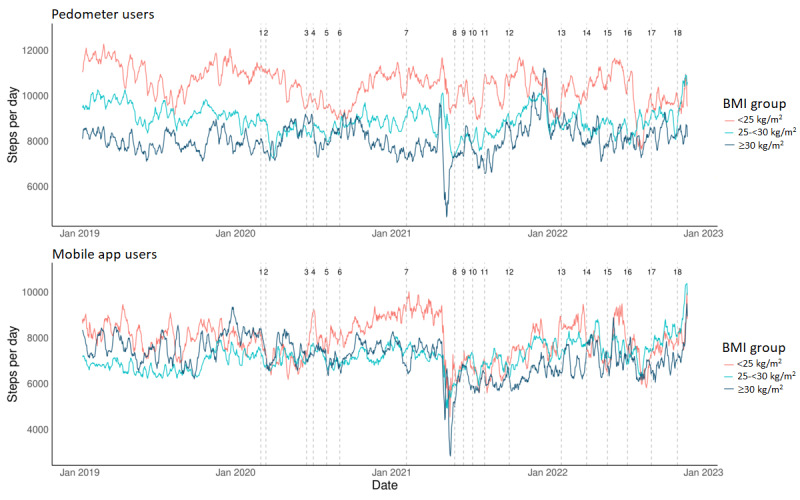
The 7-day moving average of daily step count for groups of normal weight, overweight, and obesity within the studied period covering 1 year before COVID-19 (T0: January 1, 2019, to February 28, 2020) and the 3 COVID-19 waves (T1-T18: February 29, 2020, to February 28, 2023). Details on the 19 time points reflecting the implementation and lifting of restrictions are found in Table 1. Data from pedometer and mobile app users are presented separately.

A significant effect of BMI (*F*_2, 362.63_=4.210; *P*=.02) was shown on average daily step count in the studied time period (Figure 4). Findings indicated that the normal weight group had an average of 1191 and 1557 daily steps more than the overweight (*P*=.04) and obesity (*P*=.02) groups, respectively. No significant overall differences in daily step count were revealed between the overweight and obesity groups (*P*=.85). Although no significant interaction effect was found between BMI and device type (*F*_2, 507.93_=1.459; *P*=.23), post hoc comparisons showed noteworthy differences between BMI groups using different devices (Figure 4). For instance, among mobile users, the normal weight group showed the highest fluctuations, with the different phases revealing significant decreases in step count following implementation of restrictions for wave 1 (–1276 steps compared to pre–COVID-19; *P*=.02) and a trend for wave 2 (–1070 steps compared to pre–COVID-19; *P*=.07), while no significant changes were recorded at wave 3 compared to the pre–COVID-19 period. At the end of each wave, the normal weight group recovered similar (slightly but not significantly higher) pre–COVID-19 values (Figure 4). For mobile users with overweight and obesity, less variations in step count were recorded with time. Specifically, the overweight group recorded an initial significant decrease in step count at the start of wave 1 (–691 steps compared to pre–COVID-19; *P*=.02) with no further changes until values significantly increased at the last phase of wave 3 when all restrictions were waived (+1744 step compared to pre–COVID-19; *P*<.001). The group with obesity only recorded a slight decrease of 769 steps at the start of wave 1 (compared to pre–COVID-19; *P*=.06) that persisted and became significant during wave 2 (–1259 steps compared to pre–COVID-19; *P*=.005). Slight increases in step count were seen for the group with obesity toward the end of the studied period, where daily step count regained their pre–COVID-19 values (*P*=.99; Figure 4). For pedometer users, the normal weight group recorded an initial significant decrease in values at the start of wave 1 (–1398 steps compared to pre–COVID-19; *P*=.001) and another larger significant decrease at the start of wave 2 (–1858 steps compared to pre–COVID-19; *P*<.001; Figure 4). After wave 2, values remained significantly lower until T17 (–1157 steps compared to pre–COVID-19; *P*=.02), and at the last studied period (T18), 1124 less steps were still noted compared to pre–COVID-19 (*P*=.08). The overweight group presented a similar pattern of change when compared to the pre–COVID-19 period. At the start of waves 1, 2, and 3, decreases of 995 steps (*P*=.002), 1145 steps (*P*=.002) and 1088 steps (*P*=.009) were respectively noted for the overweight group; however, an increase in values was also observed at the end of each wave with similar values noted at the end of the studied period as compared to pre–COVID-19 (Figure 4). Finally, no significant variations in step count were revealed throughout all waves for the group with obesity using the pedometer. Please refer to Tables S2 and S3 in Multimedia Appendix 1 for the average daily step count of each of the 19 periods studied for pedometer and mobile users, respectively, in all subgroups.

### Qualitative Results

#### Overview

This study explored the experiences of 9 participants regarding their PA habits during the COVID-19 pandemic. Participants provided insights into the impact of the pandemic on their PA routines, health behaviors, and motivations. The thematic analysis identified several key themes that were consistent across the transcripts. The pandemic had a significant impact on participants’ PA behaviors, perceptions, and preferences.

#### The Importance and Awareness of PA

All participants acknowledged the importance of PA for both physical and mental health. Many participants emphasized that exercise is vital for managing stress and maintaining overall well-being, especially during stressful times like the COVID-19 pandemic.

It’s very important to me, especially, you know, for stress relief. PA makes me feel better.

Participant 1, Male, 30-39 years old

It’s vital for keeping your body and mind in good shape ... in these times, it’s even more important for me.

Participant 4, Female, 30-39 years old

PA helps to reduce stress.

Participant 5, Male, 40-49 years old

Very important to me and to everybody. The benefits, for example, nowadays, with everybody being full of stress, walking helps relief that.

Participant 8, Female, 40-49 years old

While all participants acknowledged the importance of PA, there were differences in how they prioritized exercise. Several participants, particularly those who had prepandemic gym routines, viewed PA as a key part of their lifestyle. Others, however, indicated that their exercise habits were less structured during the pandemic due to limited time or motivation.

#### Disruptions to Routine and Shifting to Alternatives

The pandemic led to significant disruptions in participants’ usual exercise routines. A total of 8 (89%) participants reported difficulty maintaining regular PA due to gyms closures, restricted access to outdoor spaces, and limited group fitness classes. Many of those participants transitioned to home-based exercises, including using fitness apps, virtual classes, and simple home exercises such as walking and rope jumping. However, limitations like space, lack of equipment, and safety concerns impacted the intensity and variety of exercises. The most notable difference was that participants who had relied heavily on gyms and group fitness classes before the pandemic, such as cycling and CrossFit, experienced a more significant disruption in their PA habits. In contrast, participants who engaged in outdoor activities, such as walking or running, were somewhat less affected.

Before COVID, I used to go to the gym almost every day. After restrictions started, it became difficult to continue my routine.

Participant 2, Female, 40-49 years old

I couldn’t go to the gym during the lockdown, and gyms were closed, so I had to stop everything.

Participant 3, Male, >50 years old

My usual routine was walking and cycling, but with the restrictions, I had to adjust to home-based workouts.

Participant 6, Female, >50 years old

COVID impacted my PA a lot. All gyms closed, and I was stuck at home.

Participant 9, Male, >50 years old

I started doing home workouts with an app. It helped me stay active, even if it was hard sometimes.

Participant 1, Male, 30-39 years old

I found some virtual classes and YouTube tutorials. I joined a few online workouts but did mostly walking.

Participant 4, Female, 30-39 years old

During the pandemic, I worked out at home mostly, with some virtual classes. It was a bit hard to stay motivated at times.

Participant 5, Male, 40-49 years old

I was doing indoor exercises with an app. It was quite easy since I had enough time at home.

Participant 9, Male, >50 years old

#### Impact of COVID-19 on Lifestyle and Sedentary Behavior

A total of 7 (79%) participants reported an increase in their sedentary behavior during the pandemic. Working from home, limited outdoor activities, and lockdowns led to an overall reduction in physical movement, contributing to a more sedentary lifestyle for some. Participants noted spending longer hours in front of screens, either working or watching TV, which led to a more sedentary lifestyle. Participants who had previously been more active reported a noticeable decrease in PA, with some struggling to maintain their prepandemic routines. On the other hand, 2 (22%) participants who were less active before the pandemic reported that the lockdowns did not significantly alter their sedentary behaviors.

I didn’t move much during COVID. Most of my time was spent on my bed, working and watching TV.

Participant 5, Male, 40-49 years old

I found myself sitting for long periods while working from home. I wasn’t as active as before.

Participant 7, Male, 30-39 years old

During the lockdown, I stayed at home most of the time, and there were no real opportunities for exercise.

Participant 9, Male, >50 years old

Sedentary behavior increased during the pandemic ... I was mostly indoors, working, and didn’t get as much exercise as I wanted.

Participant 2, Female, 40-49 years old

#### Motivation and Support System

Motivation was a key factor in maintaining regular PA during the pandemic. A total of 6 (67%) participants reported relying on external sources for motivation, such as family members, social media, or online fitness communities to continue exercising. However, 3 (33%) participants struggled with maintaining consistency due to isolation, lack of support, or insufficient motivation. Participants who had a structured workout routine before COVID-19 were often able to maintain higher levels of activity during the lockdown.

Social media really helped me stay motivated. I followed a few trainers and exercise groups that kept me going.

Participant 3, Male, >50 years old

My family was supportive. They helped me stick to my routine. My wife did exercises with me at times.

Participant 8, Female, 40-49 years old

I tried to stay motivated, but it was hard at times. I had to find ways like using apps and following trainers online.

Participant 4, Female, 30-39 years old

Sometimes I felt bored, and that’s when I used YouTube videos or watched football to keep me motivated.

Participant 8, Female, 40-49 years old

#### Perceptions of Media and Communication During COVID-19

The media played an important role in shaping participants’ perceptions of COVID-19 restrictions and the importance of PA. A total of 8 (89%) participants mentioned that the media played an important role in shaping their perceptions of COVID-19 restrictions and the importance of PA. Social media influenced many participants by providing inspiration for home workouts, although some expressed feeling overwhelmed by conflicting advice on exercise and health.

The media kept reminding us how important it is to stay active even at home. That kept me going.

Participant 3, Male, >50 years old

Social media was very influential. I saw many people post about their home workouts and how to stay healthy. That motivated me to try new things.

Participant 9, Male, >50 years old

The media coverage was helpful in showing us what exercises we could do at home to stay active during the pandemic.

Participant 2, Female, 40-49 years old

Media has helped to remind us about the importance of being active during COVID. But sometimes it was overwhelming and confusing.

Participant 8, Female, 40-49 years old

#### Barriers to PA During the Pandemic

Barriers such as lack of space, absence of equipment, and restrictions on outdoor activities were significant obstacles for participants trying to maintain regular exercise during the pandemic. A total of 7 (78%) participants reported facing significant barriers to maintaining PA during the pandemic. These barriers included lack of space, absence of equipment, restrictions on outdoor activities, and psychological factors like reduced motivation or feeling overwhelmed by the stress of the pandemic. Those with access to outdoor spaces or home gyms had fewer barriers compared to those living in smaller apartments or with limited space. Additionally, participants who faced health issues or stress had more difficulty maintaining an active lifestyle during the pandemic.

I was missing my usual gym routine ... at home, I didn’t have the right equipment, and the space was limited.

Participant 5, Male, 40-49 years old

The biggest barrier for me was not being able to go outside or go to the gym ... I didn’t have enough space or equipment at home.

Participant 7, Male, 30-39 years old

It was hard to get motivated at home. And with all the stress of the pandemic, sometimes I just didn’t feel like working out.

Participant 4, Female, 30-39 years old

The restrictions on outdoor activities were a big problem. It really affected my PA levels.

Participant 8, Female, 40-49 years old

#### Changes in PA Behavior After Lifting of Restrictions

As COVID-19 restrictions were eased, 6 (67%) participants expressed a desire to return to their previous exercise routines, such as going back to the gym or engaging in group activities. However, 3 (33%) participants were still hesitant, preferring home-based workouts or smaller, less crowded exercise options due to lingering safety concerns.

Now that restrictions are lifted, I prefer going back to the gym. I missed the group classes.

Participant 6, Female, >50 years

I am still not comfortable going back to gyms. I prefer to continue exercising at home for now.

Participant 4, Female, 30-39 years old

I would like to use the gym, but you know, I have a gym at home. And the gym facilities outside are too expensive.

Participant 8, Female, 40-49 years old

I’ve started going back to the gym, but I am more cautious about the crowds. I prefer small group classes now.

Participant 9, Male, >50 years old

#### Reluctance to Return to Prepandemic Routines

The long-term impact of COVID-19 on participants’ PA habits was mixed. A total of 5 (56%) participants planned to continue with home-based exercises and more flexible routines post pandemic. Conversely, 4 (44%) participants were uncertain about how to reintegrate into their prepandemic activities. The pandemic also led some to reassess their health and fitness priorities. Mainly, those who had struggled to stay active during the pandemic showed uncertainty about returning to high-intensity or group activities.

I think after COVID, I will stick to some home exercises. It’s more convenient for me.

Participant 3, Male, >50 years old

I don’t think I will return to the same routine ... maybe I will walk more, but I don’t see myself going back to the gym regularly.

Participant 5, Male, 40-49 years old

After all of this, I plan to keep working out at home. It fits my lifestyle better.

Participant 6, Female, >50 years old

I’ve been working out more at home during COVID, and I’ll probably continue that for now.

Participant 9, Male, >50 years old

### Triangulation of Results

The use of methodological triangulation allowed for the integration of quantitative and qualitative strands, strengthening the interpretation of results. Patterns observed in step count data (such as declines during restriction phases, subgroup differences, and device-related discrepancies) were consistent with themes identified in interviews regarding disruptions to PA routines, motivational challenges, and adaptations to home-based exercise. Data triangulation further enriched the analysis by contrasting findings across devices (pedometer vs mobile phone) with participants’ perceptions. This combined approach provided a more comprehensive understanding of PA behaviors during the COVID-19 pandemic and increased the rigor of the conclusions.

Increased sedentary behavior was noted in our analyses, linked to lockdown restrictions, working from home, and limited outdoor activity. Motivation emerged as a key factor in maintaining activity, with external support from social media and family playing a significant role. These qualitative insights were reflected in the quantitative data, which indicated that engagement with online communities helped sustain exercise habits.

Barriers such as limited space, lack of equipment, and psychological factors were consistent across both data sources. Qualitative findings further highlighted how these barriers were exacerbated by stress and mental health challenges, offering a deeper context to the quantitative results. Post pandemic, participants expressed mixed intentions about returning to prepandemic routines, with some preferring home-based workouts, which was also reflected in the quantitative data.

The quantitative finding that wave 2 produced the largest declines in step count, especially among older adults, was echoed in interview data: participants described heightened fear and caution, reporting that “with all the stress of the pandemic, sometimes I just didn’t feel like working out” (Female, 30-39 years old). Similarly, discrepancies between pedometer and mobile users were contextualized by participants’ reports of not always carrying phones during exercise or preferring home-based workouts, which may have reduced recorded steps (“I was doing indoor exercises with an app. It was quite easy since I had enough time at home” [Male, >50 years old]). On the other hand, while female mobile users recorded fewer steps than male users in the pre–COVID-19 phase, some women reported higher motivation after returning to work, which corresponded with the observed increase in their step counts in wave 3 (“My family was supportive. They helped me stick to my routine” [Female, 40-49 years old]). These examples show where the qualitative themes supported or nuanced the quantitative results, highlighting both convergences and divergences.

The integration of findings through triangulation enhanced interpretive validity and underscored the value of mixed methods approaches in capturing both behavioral trends and contextual complexities. This convergence of evidence supports a more holistic understanding of PA adaptation during public health crises and informs future interventions that address both structural and psychosocial determinants.

## Discussion

### Main Findings

This study examined the impact of COVID-19 phases of restrictions on PA levels as measured by daily step count during the entire pandemic period spanning over the 3 waves. Results offer valuable insights into behavioral adaptations to restrictive measures while considering the influence of sex, age, nationality, and BMI, as well as the impact of the device used to track step count. Generally, the findings confirm significant declines in daily step count at the implementation of restrictions within each COVID-19 wave followed by a recovery of values toward the end of each wave. The latter is consistent with previous reports focusing on the first COVID-19 wave in Qatar [20] and elsewhere using community-based longitudinal interventions [34,35]. Barriers reported by participants included lack of space, absence of equipment, restrictions on outdoor activities, and psychological factors, such as reduced motivation, stress, or feeling overwhelmed.

The most pronounced decreases in PA were observed at the second wave, coinciding with the arrival of the Omicron variant in Qatar [36]. This aligns with reports of peak severity, criticality, and fatality in locally confirmed cases between March and May 2021 [37]. Heightened public fear and/or perceived risk, potentially leading to stricter adherence to restrictions, seemed to act as important barriers to PA behavior in the given context. Indeed, in addition to several barriers reported by interviewed participants, the stress of the pandemic and relative caution came up as limiting factors (“... and with all the stress of the pandemic, sometimes I just didn’t feel like working out”; “... but I am more cautious about the crowds. I prefer small group classes now”). This interpretation might indicate that severity, criticality, and fatality of cases strongly advertised on social media platforms and news outlets might have played a critical role in PA level, rather than the reported number of daily confirmed cases that peaked at a different time. Additionally, the period between April 12 and May 12, 2021, characterized with the most severe drops in daily step counts, also coincided with the month of Ramadan, where Muslims fast from dawn to sunset and naturally decrease their daily steps [38]. Large vaccination campaigns and advances in case management were among the factors that favored a 95% drop in the rates of severe, critical, and fatal cases with a turning point recorded in March 2022 [37] where increases in daily step counts were noted.

A primary factor to consider in the interpretation is the discrepancy found in the patterns of change in recorded steps between pedometer and mobile phone users. Pedometer users recorded an average of 690 daily steps more than mobile users, consistent with previous literature. A previous study showed that step count was 12% lower in iPhone users as compared to pedometer users [39]. Although underestimations seen in mobile devices are mainly attributed to noncarrying time that is influenced by the setting or context (eg, being at home or outside), other factors are worth considering. Mobile carrying habits could explain sex differences found between device groups, as women might tend to favor carrying their phones in their bags and men in their pockets [39]. Additionally, pedometers are dedicated to activity tracking unlike mobile phones, and the simple fact of carrying one can alter behaviors. Findings from a previous study on the SIH population indicated that pedometer users were significantly more committed to uploading their data and presented higher retention to the program as compared to mobile users [30]. Authors suggested that offering a free pedometer might have enhanced feelings of responsibility and motivation toward the program [40]. Finally, mobile phones fail to account for steps during activities where it is impractical to carry a phone (eg, gym training or playing team or racket ball sports) [41] which could be favored by younger groups. A recurrent observation from the qualitative analysis is that most PA routines were shifted to home-based exercises (ie, “My usual routine was walking and cycling, but with the restrictions, I had to adjust to home-based workouts”; “I started doing home workouts with an app”; “I found some virtual classes and YouTube tutorials”; “During the pandemic, I worked out at home mostly, with some virtual classes”; “I was doing indoor exercises with an app”). It is reasonable to assume that tracking activity within a limited space at home is less practical with a mobile phone as compared to a dedicated activity tracker.

In this study, differences in step count between pedometer and mobile users varied from 609 to 1264 steps according to the studied phase, with the largest differences noted toward the end of all restrictions (ie, wave 3) where 100% of employees were back to work. These observations further confirm that steps recorded by mobile phones can easily underestimate actual daily step counts in certain contexts. Moreover, pedometer users might also represent the most motivated and health-conscious portion of the population. Regardless of the device used to track steps, results should be interpreted with caution, as findings could be conflicting when trying to compare data from pedometer and mobile over an extended period of time among different subgroups, each presenting specific characteristics that might either enhance or hinder step count. These findings show that while device-driven data present an increased objectivity as compared to self-reported measures, bias in activity tracking still exists based on the presented limitations.

Overall, the qualitative analysis points toward a reluctance to go back to pre–COVID-19 PA routines and shifting to exercise within the home context (ie, “I think after COVID, I will stick to some home exercises. It’s more convenient for me”*;* “I don’t think I will return to the same routine ... maybe I will walk more, but I don’t see myself going back to the gym regularly”*;* “After all of this, I plan to keep working out at home. It fits my lifestyle better”*;* “I’ve been working out more at home during COVID, and I’ll probably continue that for now”)*.* While this study examines perceptions of a population that is considered health-conscious, it reveals long-term or lasting implications of the pandemic on behaviors and perceptions, where leaving the house or being active in outdoor settings are less favored. This shift in behavior, pointing to a tendency to remain within a confinement “state of mind,” could have significant implications for physical and mental health, especially with the emergence of the greater role of outdoor compared to indoor PA [42]. It is important to note, however, that while interpretations of potential long-term effects are limited to November 2022, this study is not able to report and/or speculate on any longer-term impacts on behavior beyond the studied time range. Findings do not represent current or ongoing trends and future research using more recent data is needed to determine whether observed changes persist over time.

While the literature is well supported by evidence on the effects of sex, age, and BMI on step count, or more recently on the impact of COVID-19 restrictions on step count, potential interactions between both categories of factors remain limited. In addition, discrepancies between data obtained from mobiles and pedometers add a layer of complexity to the interpretation that was not previously addressed in this context. Understanding these interactions is important to develop appropriate interventions and policies targeting the most vulnerable subgroups. Our findings point toward larger significant variations/fluctuations in step counts through restriction phases among male (compared to female) individuals, pedometer users (compared to mobile users), and normal weight participants (compared to individuals with overweight and obesity). At the pre–COVID-19 phase, these subgroups presented higher step count and were more affected by (or sensitive to) restrictive measures, which is in line with previous findings on Qatar’s first COVID-19 wave [20]. Interestingly, male individuals, older individuals, and normal weight groups using pedometers did not recover their initial pre–COVID-19 step count (ie, –708, –768, and –1124 steps, respectively), while female individuals, younger individuals, and those living with overweight and obesity recovered their values recorded using pedometers. In contrast, all subgroups using their mobile regained their initial step count, and most presented significantly higher values at the end of wave 3 compared to pre–COVID-19, which is a novel result emerging from the inclusion of 2 assessment devices in this study.

In sum, the use of mobile apps to track daily steps warrants a deeper interpretation of the exact use of mobile during home confinement and possible PA opportunities. The perceptions of younger vs older individuals, and female vs male, are crucial in explaining discrepancies. Different patterns of home and outdoor activities and varying preferences for gym, walking, and other sports/exercises might explain differences between device users during this phase. Notably, women were possibly more occupied at home with childcare and household responsibilities and may have been more positively affected by the “return to work” at the end of the third wave, which is reflected in a substantial increase in their step counts at this time.

The triangulation of both qualitative and quantitative results reinforces these insights, with qualitative data providing deeper context on how different factors (eg, motivation, support systems, or media influences) shaped participants’ PA behaviors during the pandemic. Ultimately, qualitative findings point at an important role of media and social media in favoring active lifestyles during periods of pandemic (“The media kept reminding us how important it is to stay active even at home”; “Social media was very influential. I saw many people post about their home workouts and how to stay healthy. That motivated me to try new things”; “The media coverage was helpful in showing us what exercises we could do at home to stay active during the pandemic”). It seems that remaining active was a common concern on social media that played an important motivating role; however, it was also perceived as overwhelming and confusing (“Media has helped to remind us about the importance of being active during COVID. But sometimes it was overwhelming and confusing”). Clear instructions and content on PA and exercise are essential during such periods and should be made available from reliable sources.

Finally, findings could be interpreted from the perspective of the social cognitive theory (SCT), which focuses on the reciprocal interaction between personal, behavior, and environmental factors [43,44]. According to this theoretical framework, the reduction in daily step count seen during restrictive phases or even during Ramadan supports the impact of environmental barriers on individual and collective behavior. Conversely, the gradual recovery of PA after the lifting of restrictions demonstrates the adaptive ability of motivated individuals to revert to usual PA as environments became more supportive. This goes in line with the SCT concept that environmental facilitators favor self-regulatory behavior. The characteristics of the studied population (eg, consistent engagement in tracking and uploading step count during the COVID-19 period and knowledge on the benefits of PA for health), which is likely to possess higher levels of self-efficacy as compared to the general population of Qatar, reflect an important determinant of PA behavior. Return to normal PA behavior with efforts to exercise from home show behavioral resilience throughout the pandemic in the sample studied. The reliance on social media for motivation and guidance, highlighted in the qualitative findings, aligns with SCT’s concept of observational learning, where individuals learn behaviors through exposure to peer or media models. The availability of peer activity, health promotion campaigns, and online guidance for exercise during the COVID-19 have likely reinforced participants’ motivation, therefore providing behavioral models at a time when usual opportunities were limited [45]. Overall, this interpretation is consistent with previous studies showing that access to supportive environments and modeled behaviors play an important role in sustaining PA during disruptive events [46]. Future research should explicitly measure SCT constructs such as self-efficacy, outcome expectations, social support, and observational learning to identify correlates and predictors of resilience or vulnerability in PA behavior [47], and to provide stronger public health interventions based on theoretical foundations.

### Limitations

In addition to the main limitation noted earlier in the step count bias associated with the use of pedometers and mobile phones (eg, noncarrying time, carrying habits, type of activities, and settings), several other limitations should be acknowledged. While the exclusion criteria applied for this study are necessary for the analysis, the retained sample comprised health-conscious individuals who continued tracking their step count through the entire COVID-19 period and regularly uploaded their activity data. Therefore, the studied sample differs from the general population of Qatar and potentially from the initial SIH cohort. A previous SIH study on 16,711 participants reported a 50% retention rate from 2012 to 2019 and a 16% adherence rate to daily data uploading [30]. The SIH is a self-managed and voluntary program designed to increase PA among the adult population of Qatar. The program has already proved its effectiveness in increasing average daily step count at the 12th month of enrollment compared to baseline data [28], that may represent more closely PA levels of the general population of Qatar. Together, this information suggests that the requirement for continuous valid step data across the 3 COVID-19 waves likely produced a subgroup of the most active participants characterized by the highest retention and adherence to the program. These factors limit the generalizability of our findings to the wider population of Qatar and should only represent the most active portion of the population. Moreover, it is important to consider the unique environmental and cultural context of Qatar, which may further limit the generalizability of the results to other countries outside of the Gulf Peninsula region. Extreme summer temperatures, often exceeding 40 °C, can substantially restrict outdoor PA and may have influenced step count at both baseline and periods of COVID-19 restrictions. Cultural norms, including gender-specific expectations for outdoor activity and preferences for indoor vs outdoor exercise, further shape patterns of PA in the studied population of Qatar.

A further limitation is that participants were recruited for the interviews through random selection rather than purposive sampling informed by device type, activity level, or BMI. While this strategy minimized researcher bias and still yielded diversity in age and sex, it may have missed key perspectives. The interviews’ small sample size (n=9) reached thematic saturation for the main themes identified, but limits the depth of subgroup-specific insights. Future studies could purposively recruit participants based on characteristics identified in the quantitative strand to strengthen the integration of mixed methods findings. Although participants’ narratives sometimes referred to their use of social media for exercise motivation, no social media tracking or collection of identifiable data occurred. This reliance on self-reported perceptions avoids privacy risks but also limits the depth of analysis on media use.

### Conclusions

The findings from this study emphasize the need for caution when interpreting step count data from mobile devices for research purposes, as measurement accuracy may vary by context, individual factors (eg, age, sex, and BMI), user behavior, and preference of certain types of activities. With the advancements of wearable multisensor devices, future studies should account for potential bias and limitations associated with the use of mobile phones and pedometers as PA data collection tool. The COVID-19 pandemic has taught us a lesson supported by the data of this study: developing tailored interventions should aim to encourage outdoor activities after confinement periods and provide reliable social media sources for PA and exercise across diverse populations. It is crucial to consider the contextual (eg, severity, criticality, and fatality of cases) and personal factors (eg, psychological, age, and sex) that influence PA behavior during public health crises such as the COVID-19 pandemic, where long-term consequences are observed in PA behaviors.

## Data Availability

All data generated or analyzed during this study are included in this published article and its supplementary information file.

## Appendix

Multimedia Appendix 1 Tables S2 and S3 present the mean ± SD of daily step counts for pedometer and mobile users across population subgroups before COVID-19 (T0) and during the 3 pandemic waves (T1-T18), 2019-2023.

## Abbreviations

PA: physical activity
SCT: social cognitive theory
SIH: Step Into Health program
