# Supplementary material for: Longitudinal Analysis of Variations in Daily Step Counts and Long-Term Implications of COVID-19 Waves and Restriction Phases in Qatar’s Step Into Health Program: Mixed Methods Study
Source: JMIR Public Health Surveill. 2026 Mar 23;12:e76860. doi: 10.2196/76860 (PMC13054218; doi:10.2196/76860)
Supplement: Multimedia Appendix 1 [file publichealth_v12i1e76860_app1.docx]

***Supplementary file***

**Table S2.** Mean and standard deviation of step count values for pedometer users for each sub-group of the population within the studied period covering one year before COVID-19 (T0: 01 January 2019 to 28 February 2020) and the three COVID-19 waves (T1-T18: 29 February 2020 to 28 February 2023).

|  | **Age (years)** | | **BMI (kg·m^-2^)** | | | **Sex** | |
| --- | --- | --- | --- | --- | --- | --- | --- |
| **Time^1^** | **≤ 50** | **> 50** | **≤ 25** | **>25 to <30** | **≥ 30** | **Female** | **Male** |
| **T0** | 9644±7402 | 9607±5879 | 10992±9125 | 9293±5140 | 7965±3969 | 9514±7531 | 9649±6474 |
| **T1** | 9707±7253 | 9240±5242 | 11072±8098 | 8968±5267 | 7703±3728 | 9296±6987 | 9504±6152 |
| **T2** | 8978±7226 | 9013±6763 | 10481±8355 | 8262±6386 | 8257±4820 | 9059±6942 | 8982±6985 |
| **T3** | 9407±7389 | 8650±5036 | 9851±7716 | 8429±5001 | 8814±5531 | 9774±6759 | 8797±6020 |
| **T4** | 8914±6738 | 8622±4979 | 9672±7300 | 8437±4969 | 7752±4300 | 9260±6313 | 8633±5694 |
| **T5** | 8524±5054 | 8875±4760 | 9299±5201 | 8450±4695 | 8303±4704 | 8835±4185 | 8694±5046 |
| **T6** | 9430±6363 | 9097±5119 | 10339±6646 | 8872±5220 | 8045±4253 | 9453±5554 | 9187±5701 |
| **T7** | 9935±6887 | 8840±5421 | 10557±6787 | 8885±5739 | 7600±4492 | 9659±6049 | 9169±6039 |
| **T8** | 9238±6803 | 8108±4923 | 10114±7033 | 7882±4855 | 7384±4529 | 8738±6072 | 8492±5658 |
| **T9** | 8919±6801 | 8684±5027 | 10058±5996 | 8109±4889 | 8219±7110 | 8657±5175 | 8799±5901 |
| **T10** | 8429±6073 | 8491±5195 | 9708±6031 | 8062±5224 | 7103±4761 | 8482±5107 | 8466±5607 |
| **T11** | 9791±7551 | 8486±5213 | 10326±6589 | 8537±5525 | 7795±6631 | 9028±5893 | 8946±6255 |
| **T12** | 10324±6822 | 9176±5314 | 10403±6358 | 9287±5491 | 8851±5861 | 9768±5716 | 9515±5915 |
| **T13** | 10542±5538 | 8752±4764 | 10179±5941 | 8914±4497 | 8436±4351 | 9485±5109 | 9196±5041 |
| **T14** | 10881±8059 | 8527±4509 | 10679±7865 | 8619±4442 | 8050±3952 | 10327±7469 | 8975±5440 |
| **T15** | 11841±9863 | 8416±4535 | 11099±8997 | 8530±4879 | 8072±3770 | 11930±9224 | 8913±5971 |
| **T16** | 9966±6714 | 8338±4679 | 9245±6225 | 8644±5081 | 8011±3567 | 10142±5404 | 8471±5223 |
| **T17** | 10322±5583 | 8750±4701 | 9748±5233 | 9034±5334 | 8333±3324 | 8877±4371 | 9179±5083 |
| **T18** | 9677±5850 | 9643±4917 | 9784±5188 | 10140±5553 | 8424±3604 | 10657±3912 | 9508±5236 |

^1^Time periods T0 to T18 reflecting the implementation and lifting of restrictions are detailed in Table 1.

**Table S3.** Mean and standard deviation of daily step count values for mobile (i.e., Android and iPhone) users for each sub-group of the population within the studied period covering one year before COVID-19 (T0: 01 January 2019 to 28 February 2020) and the three COVID-19 waves (T1-T18: 29 February 2020 to 28 February 2023).

|  | **Age (years)** | | **BMI (kg·m^-2^)** | | | **Sex** | |
| --- | --- | --- | --- | --- | --- | --- | --- |
| **Time^1^** | **≤ 50** | **> 50** | **≤ 25** | **>25 to <30** | **≥ 30** | **Female** | **Male** |
| **T0** | 6892±4683 | 8236±5818 | 8107±4693 | 6872±4481 | 7683±6224 | 5887±4641 | 7581±5165 |
| **T1** | 6567±4375 | 8568±6829 | 7515±4244 | 6908±4599 | 7649±7004 | 5503±4703 | 7529±5476 |
| **T2** | 6288±4810 | 8609±6630 | 6953±4563 | 7055±5117 | 7413±7088 | 6633±5577 | 7215±5666 |
| **T3** | 6796±4798 | 8837±6259 | 8460±5145 | 7476±5173 | 7253±6071 | 7015±5381 | 7666±5503 |
| **T4** | 6556±4787 | 8938±6484 | 7931±5380 | 7279±4986 | 7437±6633 | 6884±5428 | 7560±5635 |
| **T5** | 6360±4734 | 8315±5878 | 8013±5249 | 6837±4642 | 6895±6061 | 6402±4847 | 7191±5326 |
| **T6** | 6932±4556 | 8828±6338 | 8696±5314 | 7295±4865 | 7521±6083 | 6912±5394 | 7766±5347 |
| **T7** | 6914±4802 | 8644±6768 | 9126±6607 | 7196±5001 | 7258±6072 | 7220±6904 | 7653±5428 |
| **T8** | 6328±4333 | 6676±4245 | 6531±4311 | 6468±4289 | 6406±4322 | 5857±4675 | 6564±4227 |
| **T9** | 6870±4697 | 6699±4624 | 7020±4250 | 7096±5031 | 6235±4204 | 7618±6512 | 6678±4305 |
| **T10** | 6393±4554 | 6365±4158 | 6453±4271 | 6571±4634 | 6007±3991 | 6831±6301 | 6298±3938 |
| **T11** | 6618±5034 | 6623±4243 | 6568±4312 | 6924±5111 | 6060±4170 | 7103±7314 | 6536±4123 |
| **T12** | 7226±4973 | 7221±4488 | 7728±4754 | 7451±4955 | 6413±4357 | 6999±5832 | 7262±4594 |
| **T13** | 7657±5126 | 7761±5155 | 8540±5355 | 7807±5126 | 6917±4897 | 7114±7002 | 7776±4834 |
| **T14** | 7438±5347 | 7874±5108 | 7414±4064 | 7840±5327 | 7278±5832 | 5568±5163 | 7746±5233 |
| **T15** | 6992±5049 | 8412±5126 | 8303±5891 | 7503±4646 | 7208±5463 | 8333±7763 | 7496±4834 |
| **T16** | 6422±3951 | 7751±5130 | 6950±4084 | 7173±4490 | 6694±4934 | 7114±6063 | 6995±4417 |
| **T17** | 7680±4738 | 7718±4819 | 7724±3742 | 8061±4991 | 6891±4919 | 8731±7654 | 7624±4494 |
| **T18** | 8666±6316 | 8789±6326 | 8392±4115 | 9251±6842 | 7872±6258 | 9807±10044 | 8606±5821 |

^1^Time periods T0 to T18 reflecting the implementation and lifting of restrictions are detailed in Table 1.
